# Association of Pericoronary Adipose Tissue Quality Determined by Dual-Layer Spectral Detector CT With Severity of Coronary Artery Disease: A Preliminary Study

**DOI:** 10.3389/fcvm.2021.720127

**Published:** 2021-09-30

**Authors:** Yuxue Dang, Xujiao Chen, Shaowei Ma, Yue Ma, Quanmei Ma, Ke Zhou, Ting Liu, Kunhua Wang, Yang Hou

**Affiliations:** ^1^Department of Radiology, Shengjing Hospital of China Medical University, Shenyang, China; ^2^Department of Cardiology, Shengjing Hospital of China Medical University, Shenyang, China; ^3^Department of Cardiac Surgery, Shengjing Hospital of China Medical University, Shenyang, China; ^4^Department of Radiology, The First Affiliated Hospital of China Medical University, Shenyang, China; ^5^Department of Radiology, The People’s Hospital of Liaoning Province, Shenyang, China

**Keywords:** pericoronary adipose tissue, dual-layer spectral detector CT, coronary artery disease, fat attenuation index, epicardial adipose tissue

## Abstract

**Background:** Pericoronary adipose tissue (PCAT) is considered as a source of inflammatory mediators, leading to the development of coronary atherosclerosis. The study aimed to investigate the correlation between PCAT quality derived from dual-layer spectral detector CT (SDCT) and the severity of coronary artery disease (CAD), and whether PCAT parameters were independently associated with the presence of CAD.

**Materials and Methods:** A total of 403 patients with symptoms of chest pain who underwent SDCT were included. PCAT quality including fat attenuation index (FAI) measured from conventional polychromatic CT images (FAI_120kvp_) and spectral virtual mono-energetic images at 40 keV (FAI_40keV_), slope of spectral HU curve (λ_HU_), and effective atomic number (Eff-Z) were measured around the lesions representing the maximal degree of vascular stenosis in each patient. Meanwhile, overall epicardial adipose tissue (EAT) attenuation was acquired in the conventional polychromatic energy imaging.

**Results:** FAI_40keV_, λ_HU_, Eff-Z, and FAI_120kvp_ increased along with the degree of CAD in general and were superior to the overall EAT attenuation for detecting the presence of CAD. Multivariate logistic regression analysis indicated that FAI_40keV_ was the most powerful independent indicator (odds ratio 1.058, 95% CI 1.044–1.073; *p* < 0.001) of CAD among these parameters. Using an optimal cut-off (−131.8 HU), FAI_40keV_ showed higher diagnostic accuracy of 80.6% compared with the other parameters.

**Conclusions:** These preliminary findings suggest that FAI_40keV_ on SDCT may be an appealing surrogate maker to allow monitoring of PCAT changes in the development of CAD.

## Introduction

It is widely recognized that epicardial adipose tissue (EAT) plays a crucial role in the development of coronary artery disease (CAD) (1–3). Compared with EAT volume, EAT attenuation is more sensitive in showing the pathological changes of EAT, such as inhibition of adipocyte differentiation, interstitial fibrosis and microvascular proliferation (4, 5). Although EAT attenuation was associated with CAD risk factors and the presence of CAD, it was not associated with the presence of a significant coronary artery lesion (6). As an important component of EAT, pericoronary adipose tissue (PCAT) wrapping around coronary arteries secretes the inflammatory cytokines which may affect the adjacent vessel wall, and the resulting vascular inflammation leads to the formation and progress of coronary atherosclerosis (7–9). This complex interplay between vascular inflammation revealed by PCAT attenuation and coronary stenosis caused by atherosclerosis has been a focus of recent research. PCAT attenuation had been found to be associated with hemodynamically significant lesions, an increased risk of acute coronary syndrome, cardiac mortality and poor prognosis (10–13). Yu et al. found that perivascular fat attenuation index (FAI) provided incremental value to diameter stenosis for identifying hemodynamically significant lesions (11). However, this study included only patients with moderate to severe stenosis. The relationship between PCAT changes and the degree of coronary stenosis is still uncertain. Therefore, we conducted a preliminary study to investigate the association of PCAT attenuation and EAT attenuation with different stages of CAD in a large cohort of patients with chest pain.

Dual-layer spectral detector CT (SDCT) was introduced in recent years, which has shown to be highly suitable for coronary CTA in a clinical setting (14, 15). The use of spectral reconstructions such as virtual monochromatic imaging (VMI) at the low energy level of 40 keV can increase the contrast of soft tissue significantly (15–17). Effective atomic number (Eff-Z) and the slope of the spectral HU curve (λ_HU_) are important energy spectrum parameters, which can help to differentiate the tissue characteristics (18, 19). Based on the knowledge learned above, we hypothesized that the index of PCAT on SDCT may be more sensitive and accurate than conventional CT to reflect the changes of PCAT.

To the best of our knowledge, there are no reports of relationship of PCAT quality derived from SDCT with different degrees of CAD. Therefore, the current study aimed to (i) investigate the association between PCAT index derived from SDCT and severity of CAD, and (ii) explore whether the PCAT index can be used as an independent risk factor for CAD.

## Materials and Methods

### Patients

**Figure 1:**
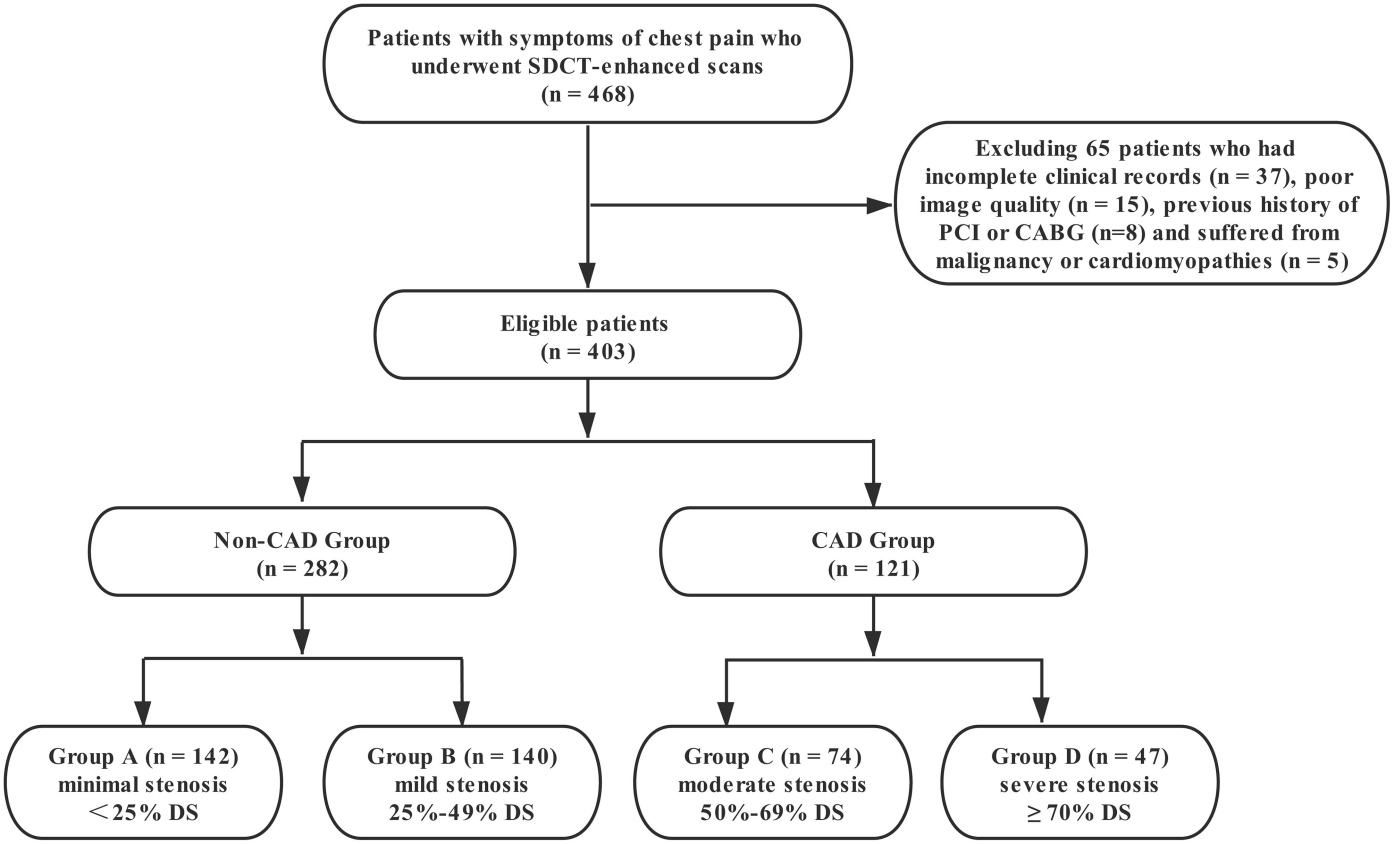
Flow diagram of patient recruitment and grouping. PCI, percutaneous coronary intervention; CABG, coronary artery bypass graft; DS, diameter stenosis.

This retrospective study was approved by the institutional review board of Shengjing Hospital of China Medical University (NO.2020PS231K). Because it was a retrospective study and the scans were performed as part of clinical routine, informed consent was exempted. Data from 468 patients with chest pain and suspected coronary artery disease who underwent coronary CT angiography (coronary CTA) on a spectral detector CT between April 2018 and July 2020 were reviewed in this study. The exclusion criteria were as follows: (1) patients with incomplete clinical records; (2) poor image quality; (3) previous history of percutaneous coronary intervention (PCI) or coronary artery bypass grafting (CABG); and (4) patients suffering from malignancy or cardiomyopathies. As a result, a total of 403 patients were included in this study (Figure 1). Among them, 121 patients were diagnosed with moderate or severe stenosis by coronary CTA, and catheter coronary angiography (CAG) was performed to further confirm the extent of lesions.

The relevant clinical data of the patients was collected by reviewing the electronic medical database. Body mass index (BMI) was calculated as weight (kg)/height^2^ (m^2^). Patients on insulin therapy or oral anti-diabetic drugs, or showing HbA1c ≥ 6.5%, fasting glucose ≥ 126 mg/dL or non-fasting glucose ≥ 200 mg/dL were considered to have Diabetes Mellitus Type 2. Systolic blood pressure ≥ 140 mm Hg, or diastolic blood pressure ≥ 90 mm Hg, or use of antihypertensive drugs was considered as an indication of hypertension. Current and previous smoking and drinking histories were reviewed. Total cholesterol (TC), triglycerides (TG), high-density lipoprotein (HDL), and low-density lipoprotein (LDL) were collected.

### CT Acquisition and Reconstruction

The coronary computed tomography angiography (coronary CTA) scans were performed on a spectral detector CT scanner (IQon, Philips Healthcare, Best, The Netherlands) using prospective electrocardiogram (ECG) gating (Step & Shoot Cardiac). The scans were performed with the following parameters: 120 kVp, 0.27s rotation time, 64 × 0.625 mm slice collimation, and the Dose Right Index set to 13. The scan trigger was centered around a physiologic cardiac phase of ventricular diastasis corresponding to 78% of the R–R interval, with a ±3% buffer used. Patients with a heart rate >70 bpm received intravenous ß-receptor blocker before the scan regularly (see Supplementary Material for detailed information).

Raw data were reconstructed using spectral iterative reconstruction algorithm with the spectral reconstruction level set to 4 (Philips Healthcare). Axial images were reconstructed at a slice thickness of 0.9 mm with an overlapping increment of 0.45 mm. The resulting spectral base image (SBI) datasets included the true conventional polychromatic (120 kVp) images along with a wide variety of spectral reconstructions. The SBI corresponding to the best cardiac phase was identified from which VMI were generated—for the purpose of image assessment, VMIs from 40 to 70 keV in steps of 10 keV were assessed.

According to the degree of coronary stenosis from CCTA and CAG, all patients were first divided into non-CAD group [< 50% luminal diameter stenosis (DS)] and CAD group (≥ 50% DS). In order to further explore the relationship of PCAT index on degrees of DS, the patients were then divided into four subgroups: Group A (minimal stenosis, < 25% DS), Group B (mild stenosis, 25%-49% DS), Group C (moderate stenosis, 50%-69% DS) and Group D (severe stenosis, ≥ 70% DS) according to the 2014 guidelines provided by the Society of Cardiovascular Computed Tomography (SCCT) (20). Furthermore, the coronary plaques of the maximal coronary stenosis were divided into calcified, non-calcified, and mixed plaques (21). The PCAT indices in the different plaque groups were further analyzed.

### Quantification of EAT and PCAT Measures

The EAT attenuation was assessed using the semi-automated software (cardiac risk assessment version 1.2.0, Siemens Healthineer, Germany). Attenuation values within −190 HU to −30 HU were considered to represent adipose tissue (13). See Supplementary Material for details on EAT attenuation measurement.

**Figure 2:**
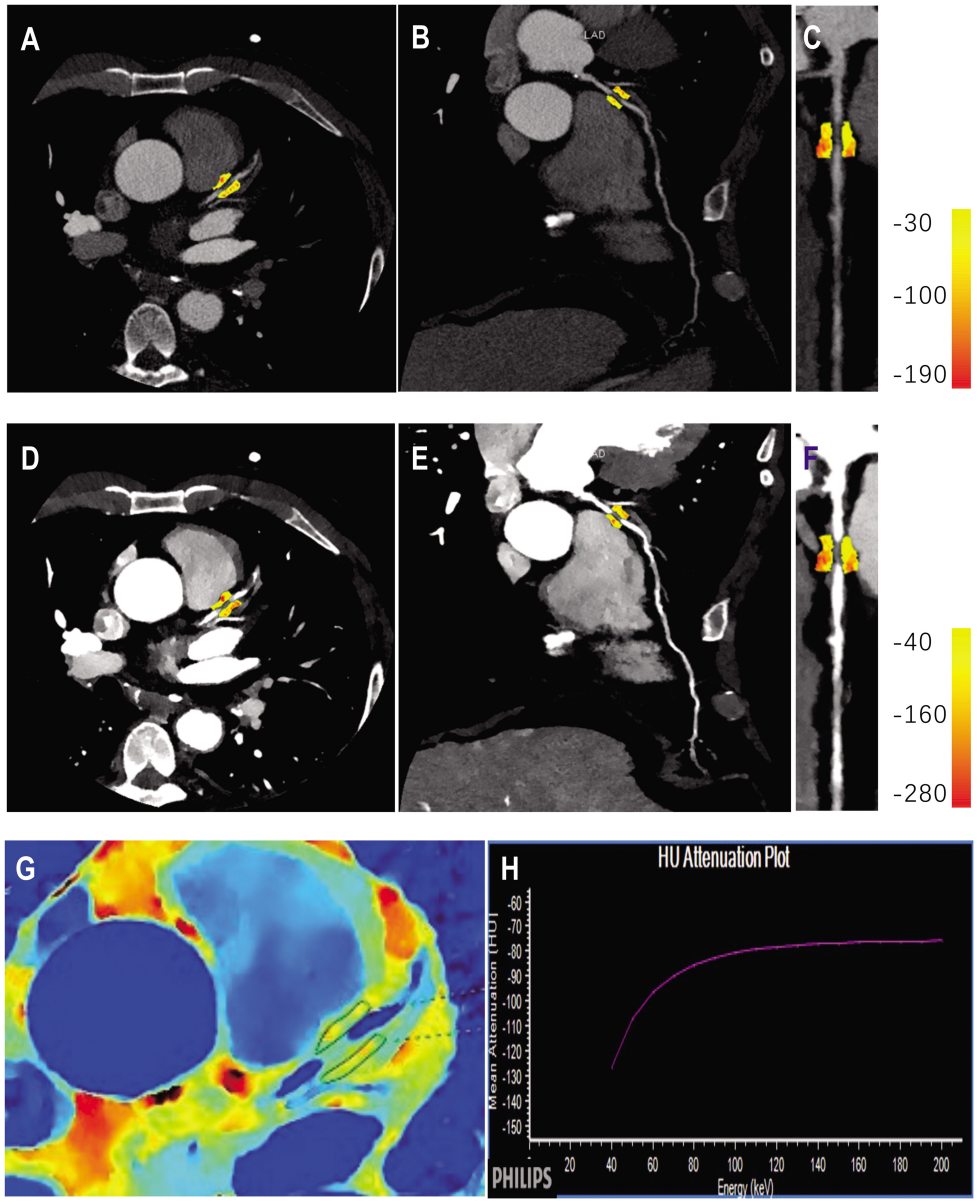
Quantitative PCAT analysis in a case example of a 56-years old male patient with significant coronary artery stenosis (severe stenosis in proximal LAD). Axial view and the corresponding curved multiplane and straightened view of PCAT of the proximal LAD in the conventional 120 kVp **(A–C)** and 40 keV VMI **(D–F)**. **(G,H)** Represented the corresponding panels of Eff-Z imaging and spectral attenuation curve, respectively. LAD, left anterior descending artery; VMI, virtual monochromatic images.

PCAT was sampled in three-dimensional layers radially outwards from the outer vessel wall and measured (7, 13). Adipose tissue was identified as all voxels with attenuation thresholds of −190 to −30 HU in conventional polychromatic energy imaging, −280 to −40 HU in VMI at 40 keV, and −220 to −30 HU in VMI at 70 keV, respectively (22). PCAT analysis was performed in the adipose tissue around the plaque that caused the maximal degree of vascular stenosis in each patient (Figure 2). FAI was measured within the predefined volume of interest at the following energy levels: conventional 120 kVp, and at spectral VMI levels of 40 keV and 70 keV using the semi-automated software (Dr. Wise^®^ Coronary Artery CT Aided Diagnosis Software V200831), reported as FAI_120kvp_, FAI_40keV_, FAI_70keV_, respectively and the slope of the spectral HU curve (λ_HU_) was calculated using the formula: λ_HU_ = (FAI_40keV_-FAI_70keV_)/30. The effective atomic number (Eff-Z) was measured using the dedicated workstation (IntelliSpace Portal Version 6.5, Philips Healthcare).

All measurements were repeated three times by a radiologist with 8 years of experience in coronary CTA and averaged in order to ensure the accuracy of the data, and the investigator was blinded to clinical information and grouping situation. In order to verify the repeatability of the PCAT measures, 60 cases were randomly selected in a blinded manner and re-measured by the same investigator after 6 weeks.

### Statistical Analysis

Datasets were analyzed using commercially available software (SPSS version 20.0, USA and MedCalc Statistical Software, version 15.2). Continuous variables were presented as means ± standard deviation. Inter-group comparisons were performed with independent–samples *t*-test or ANOVA. Welch’s *t*-test was used in data with heterogeneity of variance among multiple groups. And the LSD or Dunnett’s T3 test was used for paired comparison. Multivariate binary logistic regression was used to evaluate the association of EAT and PCAT measures with coronary stenosis. In order to compare the relative weight values of EAT and PCAT measures, each variable was standardized, and then performing multivariate logistic regression analysis to compare the standard partial regression coefficient of each variable. The receiver-operating characteristic (ROC) curve was drawn and the corresponding optimal cut-off value for predicting the presence of CAD was determined. The intra-class correlation coefficient (ICC) was used to evaluate intra-reader reliability of PCAT measurements. Generally, an ICC value ≥ 0.75 indicates excellent reliability, between 0.40 and 0.75 indicates fair to moderate reliability, and < 0.40 indicates poor reliability (23). A two-sided *p* < 0.05 was considered significant.

## Results

### Patients

There were 403 subjects included in this study. Clinical characteristics in different groups of the study cohort are shown in Table 1.

**Table 1:** Baseline characteristics of study cohort (*n* = 403).

	**All patients**	**Non-CAD**	**CAD**	***p***
	**(***n*** = 403)**	**(***n*** = 282)**	**(***n*** = 121)**	
Age (years)	57.4 ± 10.1	56.0 ± 10.6	60.5 ± 8.1	< 0.001^*^
Males (%)	229 (56.8)	145 (51.4)	84 (69.4)	0.001^*^
BMI (kg/m2)	25.5 ± 3.8	24.9 ± 3.6	26.9 ± 3.9	< 0.001^*^
**History**				
Smoking (%)	116 (28.8)	70 (24.8)	46 (38.0)	0.011^*^
Drinking (%)	98 (24.3)	58 (20.6)	40 (33.1)	0.012^*^
Diabetes mellitus (%)	121 (30.0)	78 (27.7)	43 (35.5)	0.125
Hypertension (%)	142(35.2)	86 (30.5)	56 (46.3)	0.003^*^
**Laboratory index**				
Total cholesterol (mmol/L)	4.76 ± 1.10	4.71 ± 1.00	4.86 ± 1.29	0.273
Triglycerides (mmol/L)	1.40 ± 1.02	1.33 ± 0.74	1.58 ± 1.46	0.074
HDL -cholesterol (mmol/L)	1.11 ± 0.44	1.17 ± 0.46	0.96 ± 0.36	< 0.001^*^
LDL -cholesterol (mmol/L)	2.97 ± 0.94	2.82 ± 0.86	3.33 ± 1.01	< 0.001^*^

*Compared with the non-CAD group*,

* *p < 0.05 was statistically significant*.

### Comparison of the EAT Attenuation and PCAT Parameters on SDCT in Patients With and Without CAD

**Figure 3:**
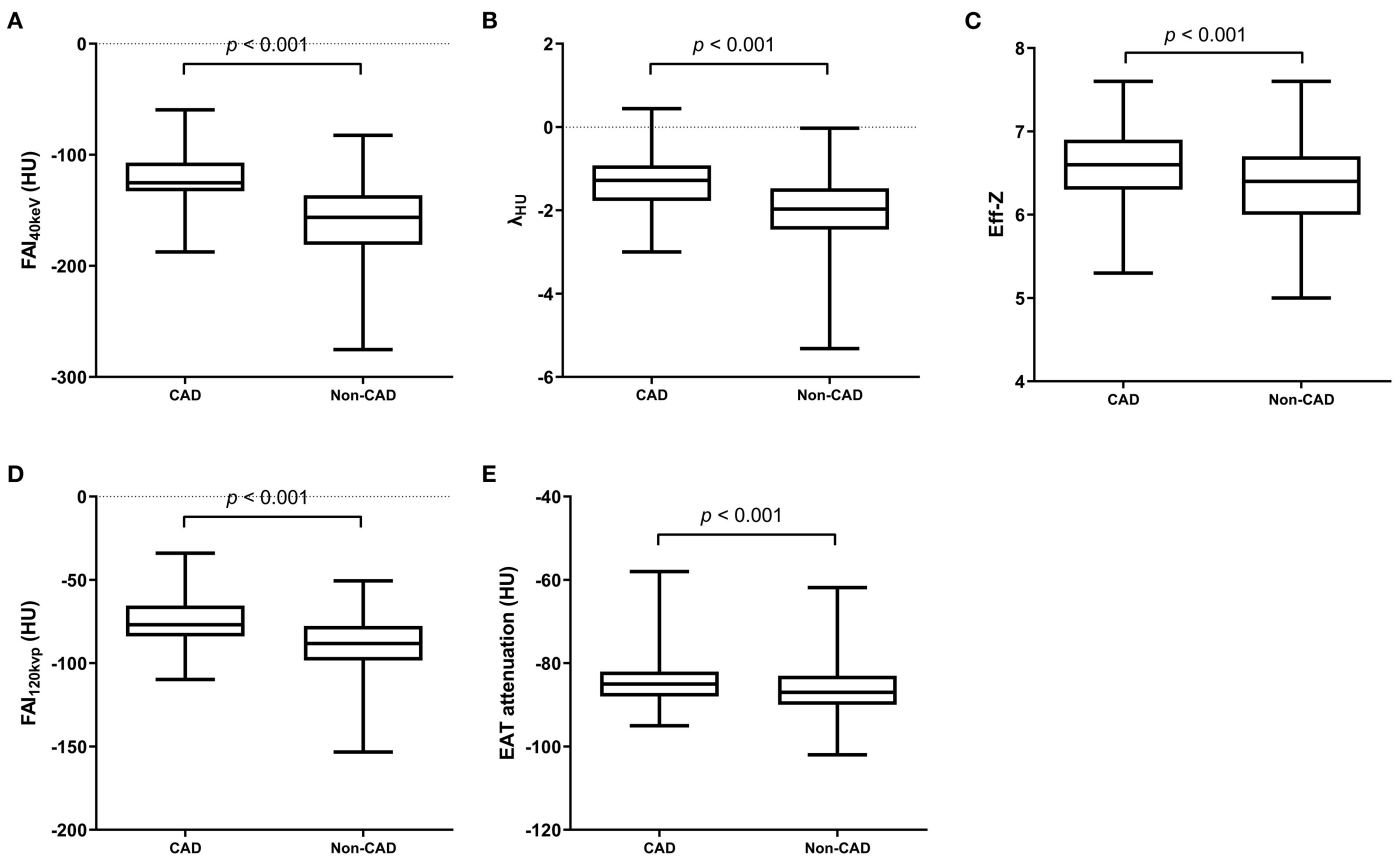
SDCT-related parameters of PCAT and EAT attenuation in CAD and non-CAD groups. FAI40keV **(A)**, λHU **(B)**, Eff-z **(C)** and FAI120kvp **(D)** and EAT attenuation **(E)** were significantly larger in patients with CAD than those in non-CAD group (both *p* < 0.001). A value of *p* < 0.05 indicated statistical significance.

All PCAT indices showed excellent reliability (ICC > 0.75) (Supplementary Table 1). The FAI_40keV_, λ_HU_, Eff-z, and FAI_120kvp_ were significantly larger in patients with CAD than those in non-CAD group (both *p* < 0.001). EAT attenuation in the two groups were also statistically different (*p* < 0.001) (Figure 3).

### PCAT Parameters and EAT Attenuation From SDCT With Relation to the Degree of Coronary Stenosis

Patients were further divided into four subgroups according to the degree of coronary stenosis, Group A (minimal), Group B (mild), Group C (moderate), and Group D (severe) (see Methods). Lesion characteristics are shown in Table 2. There were significant differences in PCAT index and EAT attenuation among the groups. In view of the overall situation, FAI_40keV_, λ_HU_, Eff-Z, FAI_120kvp_, and EAT attenuation all showed an increasing trend along with an increased degree of vascular stenosis (Table 3). Additionally, significant differences were seen in FAI_40keV_ and λ_HU_ between Groups C and D, with no significant differences seen in the other parameters of the two groups.

**Table 2:** Lesion characteristics of coronary CTA in the four subgroups.

**Characteristics**	**Group A**	**Group B**	**Group C**	**Group D**
Number of cases	142	140	74	47
**Number of lesion branches**				
Single-vessel lesion	73	50	6	7
Double-vessel lesions	46	25	7	10
Triple-vessel/ LM lesions	23	65	61	30
**Localization of the most serious lesions**				
LM	4	6	3	0
LAD	70	77	47	30
LCX	13	7	4	3
RCA	55	50	20	14
**Types of plaques characters of the maximal coronary stenosis**				
Non-calcified plaques	53	40	29	14
Mixed plaques	11	41	32	25
Calcified plaques	78	59	13	8

*Group A, minimal stenosis; Group B, mild stenosis; Group C, moderate stenosis; and Group D, severe stenosis*.

*Number of lesion branches: the presence of any plaque in the coronary artery (LAD, LCX, RCA and LM), irrespective of the degree of stenosis*.

*LM, Left main coronary artery; LAD, left anterior descending artery; LCX, Left circumflex coronary artery; RCA, Right coronary artery*.

**Table 3:** SDCT-related parameters of PCAT and EAT attenuation in different degree of stenosis groups.

	**Group A**	**Group B**	**Group C**	**Group D**	***F***	***p***
**PCAT indices on SDCT**						
FAI_40keV_ (HU)	−159.6 ± 32.5^#^	−160.9 ± 35.4^#^	−131.0 ± 25.3^*^^#^	−113.0 ± 22.5^*^	57.168	< 0.001
λ_HU_	−2.0 ± 0.8^#^	−2.1 ± 0.8^#^	−1.5 ± 0.7^*^^#^	−1.2 ± 0.6^*^	25.692	< 0.001
Eff-Z	6.3 ± 0.5^#^	6.3 ± 0.5^#^	6.6 ± 0.4^*^	6.6 ± 0.5^*^	8.187	< 0.001
FAI_120kvp_ (HU)	−89.8 ± 15.3^#^	−88.3 ± 18.4^#^	−77.7 ± 13.0^*^	−71.4 ± 14.8^*^	26.381	< 0.001
EAT attenuation (HU)	−86.5 ± 5.5^#^	−87.6 ± 6.0^#^	−83.4 ± 8.9^*^	−82.1 ± 9.5^*^	7.818	< 0.001

* *p < 0.05 vs. Group A*;

# *p < 0.05 vs. Group D*.

**Figure 4:**
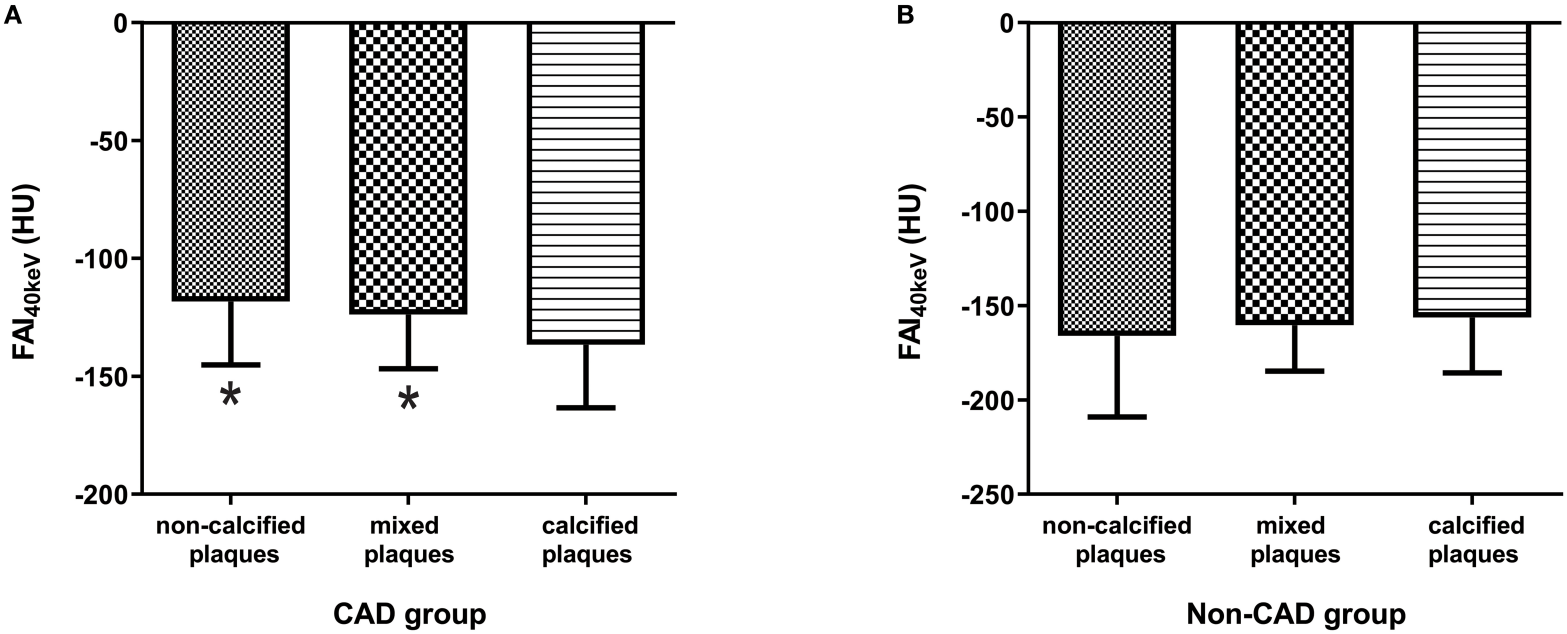
Comparison of FAI40keV (HU) based on plaque type in CAD group **(A)** and non-CAD group **(B)**. ^*^p < 0.05 compared to calcified plaques.

Furthermore, FAI_40keV_ was larger in the maximal stenosis segments with the non-calcified or mixed plaques than those with the calcified plaques in CAD group (*p* < 0.05, Figure 4A). While no significant difference was observed between the patients with non-calcified and mixed plaques. Interestingly, the opposite results were obtained in non-CAD group, which showed that FAI_40keV_ adjacent to non-calcified plaques was the lowest (Figure 4B). However, there were no significant differences in FAI_40keV_ between patients with different type of plaques.

### The Association of PCAT and EAT Measures With the Presence of CAD

On multivariate binary logistic analysis, after adjusting the conventional cardiovascular risk factors (age, gender, BMI, Diabetes Mellitus Type 2, hypertension, smoking, drinking, TC, TG, HDL, LDL) and type of plaques, FAI_40keV_, λ_HU_, Eff-Z, FAI_120kvp_, and EAT attenuation were found to be significantly associated with the presence of CAD (Table 4). It should be noted that due to the collinearity between the above-mentioned PCAT-related parameters (FAI_40keV_, λ_HU_, Eff-Z, FAI_120kvp_, and EAT attenuation), we, respectively, substituted these parameters into the regression equation for analysis. According to the standard partial regression coefficient, among these parameters, FAI_40keV_ was the most powerful independent indicator.

**Table 4:** The association of PCAT measures and EAT attenuation with the presence of CAD.

**Analyses**	**Standard partial**	**OR**	***p***
	**regression**	**(95% CI)**	
	**coefficient**		
1st: FAI_40keV_ (HU)	2.029	1.058 (1.044–1.073)	<0.001
2nd: λ_HU_	1.429	5.733 (3.514–9.352)	<0.001
3rd: Eff-Z	0.752	4.857 (2.449–9.635)	<0.001
4th: FAI_120kvp_ (HU)	1.265	1.076 (1.054–1.099)	<0.001
5th: EAT attenuation (HU)	0.688	1.100 (1.056–1.146)	<0.001

*OR, odds ratio; CI, confidence interval. p < 0.05 was regarded as indicating statistical significance*.

### ROC Analysis for Prediction of CAD

**Figure 5:**
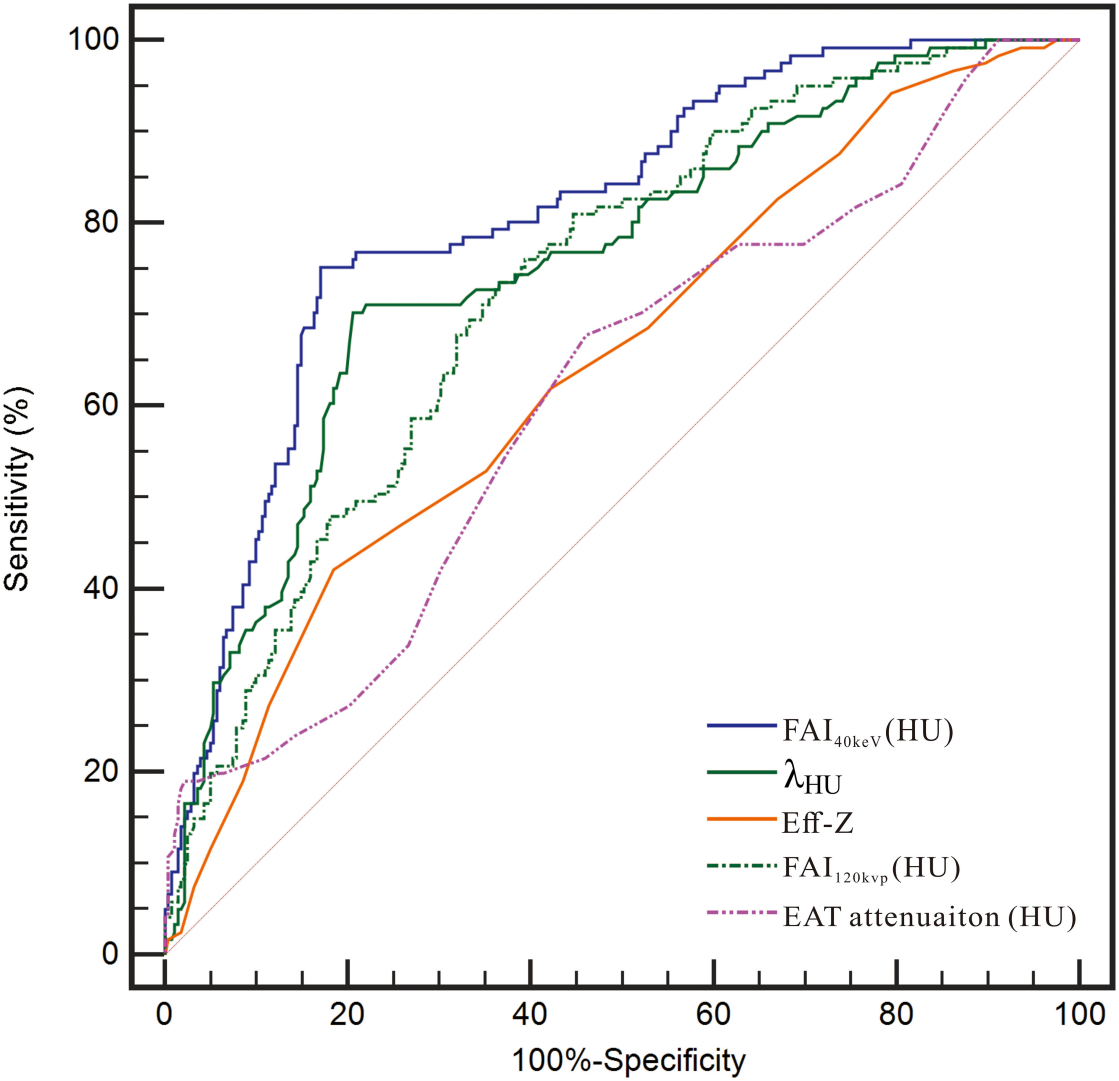
Receiver operating characteristic (ROC) curve analysis showed that compared with other parameters, the FAI_40kev_ had the highest the area under curve (AUC).

The ROC curve for FAI_40keV_, λ_HU_, Eff-Z, and FAI_120kvp_ showed them as superior predictors of CAD with the corresponding areas under curve (AUC) 0.811, 0.756, 0.646, and 0.731, respectively, which were greater than AUC of EAT attenuation (0.614) (Figure 5 and Table 5). FAI_40keV_ showed the highest AUC with the optimal cutoff value of −131.8 HU to predict CAD, with a diagnostic accuracy of 80.6% compared to the other parameters.

**Table 5:** Cut-off values of PCAT indices and EAT attenuation for the detection of CAD.

**Parameters**	**Cut-off value**	**AUC (95% CI)**	**Sensitivity (%)**	**Specificity (%)**	**Accuracy (%)**
FAI_40keV_ (HU)	−131.8	0.811 (0.769–0.848)	75.2	83.0	80.6
λ_HU_	−1.4	0.756 (0.712–0.798)	70.3	79.4	77.2
Eff-Z	6.7	0.646 (0.597–0.693)	42.2	81.6	66.0
FAI_120kvp_ (HU)	−83.0	0.731 (0.685–0.774)	73.6	63.5	66.5
EAT attenuation (HU)	−87.0	0.614 (0.564–0.662)	67.8	53.9	54.6

*AUC, area under the curve*.

## Discussion

Our preliminary findings demonstrated that FAI_40keV_ and λ_HU_ obtained from SDCT were superior to conventional polychromatic CT for detecting the pathologic changes of PCAT during the development and progression of coronary atherosclerosis. Furthermore, PCAT index on SDCT was independently associated with the presence of CAD and FAI_40keV_ was found to be the most powerful independent indictor for CAD.

To the best of our knowledge, this is the first study exploring the relationship between PCAT indicators derived from SDCT with coronary stenosis. The present study showed statistical differences in FAI_40keV_, FAI_120kvp_, and EAT attenuation between the non-CAD and CAD groups. The results are consistent with previous studies (6, 24), which found EAT attenuation was significantly higher in the CAD group than that in the non-CAD group. In the early stage of atherosclerosis, perhaps due to the expansion of adipose tissue and accumulation of lipid (25), FAI is relatively low. With the progression of coronary atherosclerosis, as there exists a complex, bidirectional interplay between the vascular wall and perivascular fat (26), pro-inflammatory cytokines released from the vessel wall diffuse directly into the surrounding peri-coronary fat the resulting perivascular inflammation promotes the adipose tissue remodeling and fibrosis (27, 28). Meanwhile, the maturity of the adipocytes and accumulation of intracellular lipids are inhibited (29–31), the adipocyte size get smaller, the lipid droplet content are reduced (10, 32), resulting in FAI increases. In the late stage of coronary atherosclerosis, fibrosis of adipose tissue could aggravate the increase in FAI. As a result, the more severe the coronary stenosis, the higher FAI level.

Furthermore, significant differences were seen in the values of FAI_40keV_ and λ_HU_ between the moderate and severe stenosis groups, with no significant differences between the FAI_120kvp_ and EAT attenuation between the two groups. Correspondingly, the AUCs for FAI_40keV_ and λ_HU_ were higher than those of FAI_120kvp_ and EAT attenuation. Based on the results, it is speculated that the spectral information from SDCT is superior to conventional polychromatic CT in detecting subtle changes in adipose tissue caused by PCAT remodeling during atherosclerosis progression. SDCT is an imaging technology introduced in recent years, which addresses the limitation of conventional polychromatic CT which assess coronary lesions only from a morphological point of view, by providing spectral results such as VMI at a wide range of energy levels (33). In addition, SDCT enables multiple quantitative results, including λ_HU_ and Eff-Z, which make it possible to perform tissue characterization (34, 35). With the capability of simultaneous, homologous and isotropic information, and not requiring any a priori scan decisions, the SDCT provides workflow benefits making it more convenient for routine clinical use. Compared with the values obtained from a polychromatic beam, the CT value under single energy can more accurately reflect the X-ray absorption characteristics of the materials (36). We suggest that the PCAT derived from single energy reconstructions at the low end of the spectrum (corresponding to 40 keV) can better differentiate the fat densities. This is consistent with prior work of Rodriguez-Granillo et al. that showed that VMI at low energy levels (40 keV) can detect differences of fat densities in different locations (22). In our work, we also observed a similar trend in λ_HU_ and Eff-Z. Since PCAT itself is a fat component, the energy spectrum curve is a curve with the back of the arch arched upwards, and the attenuation increased with higher keV. λ_HU_ in the energy range of 40 to 70 keV is negative, and it is significantly higher in the CAD group than that in the non-CAD group. λ_HU_ may be one of the markers reflecting the inflammatory state of PCAT. However, Eff-Z is not sensitive to FAI_40keV_ and λ_HU_ in reflecting pathological changes of PCAT because of its insufficient resolution. These findings highlight the potential value of SDCT in displaying fine changes of PCAT adjacent to plaques that could not be observed using conventional polychromatic CT. Our preliminary results suggest that FAI_40keV_ and λ_HU_ could be the novel surrogate image-markers reflecting the remodeling of PCAT.

Our findings also suggest that PCAT has a better ability to predict vascular stenosis than overall EAT. The possible reasons can be interpreted from the following aspects: Firstly, in terms of pathophysiology, PCAT could act adjacent coronary arteries directly contributing to the development of atherosclerosis through secretion of a large number of pro-inflammatory adipocytokines relating to energy metabolism and causing inflammation in a paracrine and vasocrine manner more than as a systemic inflammatory effect (3, 37), whereas EAT attenuation more reflects the effect of abnormal lipid metabolism on atherosclerosis, that is, it reflects its role as a visceral fat bank rather than local inflammatory effects. Additionally, when the lesion is mainly on a single coronary artery, the predictive value of global EAT attenuation for vascular stenosis may be weakened as the peri-coronary fat around normal or non-significantly stenosis vessels was relatively normal.

Marwan et al. (38) support a hypothesis of different types or activity of PCAT, the more metabolically active of which might exert local effects on the coronary vessels, thus contributing to atherogenesis. The current study showed that FAI_40keV_ was significantly higher in the maximal stenosis segments with non-calcified or mixed plaques than those with the calcified plaques in CAD cases. This may suggest that PCAT surrounding the atherosclerotic plaques with non-calcified component has higher metabolically activity. This may be related to the characteristic of highly inflamed atherosclerotic plaque, which has been discussed in detail in another separate work of our research team (39). Interestingly, the opposite results were obtained in non-CAD cases, which showed that FAI_40keV_ adjacent to non-calcified plaques was the lowest, although the differences between the groups were not statistically significant. The reason might be that low-stage inflammation causing more lipid droplets accumulated in adipocytes and an increase in cell size (40, 41) in the early stage of atherosclerotic plaque formation. Previous research (21, 42) has shown that EAT volume was significantly greater in coronary segments with non-calcified or mixed plaques compared than those with calcified plaques. This may lead to the low attenuation of PCAT adjacent non-calcified plaques. The results seemed to suggest that the effect of different types of plaque on the adjacent PCAT is not invariable with the progress of atherosclerosis.

Recent studies (10) show that FAI can non-invasively monitor the vascular inflammation and the development of CAD *in vivo*. Our results demonstrated that FAI_40keV_ was the best predictor for CAD with the optimal cut-off value of −131.8 HU. FAI_40keV_ achieved higher AUC, sensitivity and accuracy than other parameters because of its higher sensitivity in detecting adipose attenuation differences. And with the degree of atherosclerotic lesion, FAI_40keV_ tended to increase as mentioned in the above discussion, which reflected that FAI_40keV_ could detect the subtle changes of PCAT around the lesions in different stages of CAD. Monitoring changes in FAI_40keV_ during at different phases of CAD may help to better understand the mechanism of PCAT promotes atherogenesis. This may make FAI_40keV_ a new promising imaging marker to identify and monitor the course of coronary atherosclerosis. However, there are few studies on PCAT based on SDCT, and it is difficult for us to compare with other studies.

Several limitations should be noted. First, this study is only a single-center retrospective study with limited sample size. The results need to be verified in a large prospective cohort. Second, this was a cross-sectional study, with the initial state of the study subjects unknown, and a longitudinal study would be more helpful to reveal the relationship between progression of atherosclerosis and PCAT. And lastly, the optimal cut-offs of identified need further validation.

## Conclusions

Our preliminary results support the superiority of the FAI derived from spectral CT images (FAI_40keV_) along with the slope of the spectral attenuation curve (λ_HU_) over the corresponding index from conventional polychromatic CT reconstructions (FAI_120kvp_) and EAT attenuation for detecting the pathologic changes of PCAT during the development and progression of atherosclerosis. FAI_40keV_ could be a novel dynamic surrogate imaging marker of vascular inflammation and if its potential value was further verified in larger clinical trials with outcomes data, this could be a powerful indicator of a potential occurrence, development and prognostic information of a coronary lesion.

## Data Availability Statement

The original contributions presented in the study are included in the article/Supplementary Material, further inquiries can be directed to the corresponding authors.

## Ethics Statement

The study was approved by institutional review board of Shengjing Hospital of China Medical University. Written informed consent for participation was not required for this study in accordance with the national legislation and the institutional requirements.

## Author Contributions

YD, KW, and YH participated in conceiving and designing of the study. YD, XC, SM, YM, QM, KZ, and TL contributed to collecting and assembling of data. YD, XC, KW, and YH contributed to analyzing and interpreting of data. YD, XC, and YH contributed to drafting of the manuscript. All authors approved the final version of manuscript for publication.

## Funding

This study has received funding by the National Natural Science Foundation of China (Grant Nos. 82071920 and 81901741), the Key Research and Development Plan of Liaoning Province (No. 2020JH2/10300037) and 345 Talent Project in Shengjing Hospital of China Medical University.

## Conflict of Interest

The authors declare that the research was conducted in the absence of any commercial or financial relationships that could be construed as a potential conflict of interest.

## Publisher's Note

All claims expressed in this article are solely those of the authors and do not necessarily represent those of their affiliated organizations, or those of the publisher, the editors and the reviewers. Any product that may be evaluated in this article, or claim that may be made by its manufacturer, is not guaranteed or endorsed by the publisher.
