# Supplementary material for: Association of Pericoronary Adipose Tissue Quality Determined by Dual-Layer Spectral Detector CT With Severity of Coronary Artery Disease: A Preliminary Study
Source: Front Cardiovasc Med. 2021 Sep 30;8:720127. doi: 10.3389/fcvm.2021.720127 (PMC8514719; doi:10.3389/fcvm.2021.720127)
Supplement: Supplementary file 1 [file Data_Sheet_1.DOCX]

**CT acquisition protocol**

The coronary CTA scans were performed on a spectral detector CT scanner (IQon, Philips Healthcare, Best, The Netherlands) with prospective ECG gating technique. Using an 18-gauge catheter dual-tube high pressure syringe (Ulrich REF XD 2051), contrast media (Visipaque) (Iodixanol 270; GE Healthcare, Ireland) was injected into the antecubital vein with a flow rate of 4.5 ml/s (<80 kg body weight) or 5 ml/s (≥80 kg body weight) followed by a 30 ml saline flush. The total amount of contrast media was based on patient weight (patient weight × 0.8 ml / kg body weight). The scans were performed with the following parameters: 120 kVp, 0.27s rotation time, 64 ×0.625 mm slice collimation, and the Dose Right Index set to 13. The scan trigger was centered around a physiologic cardiac phase of ventricular diastasis corresponding to 78% of the R–R interval, with a ±3% buffer used. Ahead of CT examination, patients with heart rate (HR) >70 bpm were given 25-50 mg of ß-receptor blocker (Metoprolol Succinate sustained-release tablets, AstraZeneca, Sweden) orally to reduce the HR to below 70 bpm.

**Quantification of EAT attenuation**

EAT is defined as the fat depot between myocardium and pericardium and in the 120-kVp voltage, the Hounsfield units (HU) within -190 to -30 as the range of attenuation values defining fat ([1](#_ENREF_1)). EAT attenuation was quantified based on the conventional polychromatic energy images. Regions of interest (ROI) were automatically placed tracing the contour of the epicardium from the beginning of left pulmonary artery to the bottom of heart on the end diastolic images with a 10 mm interval. Partially outlined errors in the pericardial contour required manual correction. Attenuation values were calculated automatically as the mean HU value of all pixels.

**References**

1. Goeller M, Achenbach S, Cadet S, Kwan AC, Commandeur F, Slomka PJ, et al. Pericoronary Adipose Tissue Computed Tomography Attenuation and High-Risk Plaque Characteristics in Acute Coronary Syndrome Compared With Stable Coronary Artery Disease. *JAMA cardiology* (2018) 3(9):858-63. doi: 10.1001/jamacardio.2018.1997. PubMed PMID: 30027285; PubMed Central PMCID: PMC6233643.

**Table S1**

The interclass correlation coefficient (ICC) values of PCAT measures

| Parameters | intra-reader concordance |
| --- | --- |
|  | ICC (95% CI) |
| FAI_120kvp_ (HU) | 0. 844 (0.738-0.906) |
| FAI_40keV_ (HU) | 0.895 (0.825-0.937) |
| λ_HU_ | 0.863 (0.771-0.918) |
| Eff-Z | 0.775 (0.623-0.866) |
